# Importance of recognizing discordance between Alcohol Use Disorders Identification Test-Consumption (AUDIT-C) screening results and drinking reported on individual AUDIT-C questions

**DOI:** 10.1186/1940-0640-7-S1-A60

**Published:** 2012-10-09

**Authors:** Amy Lee, Kate E Delaney, Gwen T Lapham, Anna D Rubinsky, M Laura Johnson, Katharine A Bradley

**Affiliations:** 1 Department of Health Services, VA Puget Sound Healthcare System, University of Washington, Seattle, WA, USA; 2 Health Services and Development, VA Puget Sound Health Care System, Seattle, WA, USA; 3 Group Health Cooperative, VA Puget Sound, University of Washington, Seattle, WA, USA

## 

The Alcohol Use Disorders Identification Test-Consumption (AUDIT-C) is a three-item alcohol screening questionnaire where threshold scores of ≥4 for men and ≥3 for women have been associated with a higher risk of alcohol misuse. However, some response patterns yield inconsistencies: some individuals who report drinking within National Institute on Alcohol Abuse and Alcoholism (NIAAA) recommended limits on the individual AUDIT-C screen positive based on the AUDIT-C score, and some who report exceeding drinking limits on the individual AUDIT-C screen negative based on the AUDIT-C score. The objective of this study was to determine the prevalence of discordant AUDIT-C screening results and reported drinking on the individual AUDIT-C questions among men and women who reported drinking alcohol in the 2001-2002 National Epidemiologic Survey on Alcohol and Related Conditions (NESARC) (n = 26,210) and to discuss the implications of this discordance for brief interventions. Among men in the US population-based sample, 5.0% had negative screens despite reporting risky drinking, and 8.8% had positive screens while reporting drinking within recommended limits. Among women, 0.8% had negative screens despite report of risky drinking, and 17.4% had positive screens despite reporting drinking within recommended limits. Of those with negative screens, all individuals who exceeded drinking limits reported heavy episodic drinking on AUDIT-C question 3, suggesting that brief interventions for heavy episodic drinking can be offered to 5% of men and about 1% of women who do not screen positive on the AUDIT-C. Patients who screen positive on the AUDIT-C despite not reporting risky drinking can be advised explicitly about drinking limits and told that, although they are not reporting drinking above the recommended limits, some patients with similar scores develop problems due to drinking. Web-based screening and brief intervention programs offer an opportunity to provide appropriate and specific feedback to address discordant AUDIT-C response patterns.

